# Unraveling the migraine origin: is it genetics or environmental?

**DOI:** 10.1055/s-0043-1774817

**Published:** 2023-10-04

**Authors:** Mario Fernando Prieto Peres

**Affiliations:** 1 Hospital Israelita Albert Einstein, São Paulo SP, Brazil.; 2 Universidade de São Paulo, Faculdade de Medicina, Hospital das Clínicas, Instituto de Psiquiatria, São Paulo SP, Brazil.

The search for the cause of migraine makes patients' journey another headache. When one migraine trigger is identified as the only cause of patients' headaches, its resolution is self-managed easily, the dream becomes true, that is the example of alcohol induced migraines or food related headaches. 1

In the neurologist's clinical practice, this is rarely the case, patients dream become a nightmare, and the explanation model of the disease must be changed, for a disorder with genetic predisposition influenced by multiple mechanisms, environmental factors, hormonal, physical, mental, and most likely, several of the possible mechanisms associated. 2

How much migraine is genetic or environmental is a matter of continuous and long debate in the literature. Dück et al, 3 contributed to this battle when they sought to compare Menonites descendants, an excellent model of genetic isolation, versus controls in the general population living in an urban region of Curitiba, Brazil. The study meticulously collected data from both populations, documenting migraine frequency, duration, intensity, triggers, and associated symptoms.

No significant differences were found between groups, but sleep, a more diffuse headache, and depressive symptoms in controls. In a world where science is increasingly uncovering the complexity of human health, this study reminds us that while genes provide the script, the environment directs the performance. Is nurture more significant than nature? It is not the final word in the debate, nevertheless, the data presented in this study may be biased due to some limitations.

Even though Menonites have been genetically isolated, they may not be that different from controls, with their majority of Caucasians. The sample size is small, prevalence rates cannot precisely be ascertained. The population living in the urban region may be more prone to factors independent of genetic predisposition or migraine genes may not be related to factors this population is exposed to.

When evaluating the influence of predisposition in migraine, one has to keep in mind migraine genetics is complex, polygenic, with a genetic component, family history, observed in 30–60% patients. 4 Genome-wide association studies (GWAS) have been identifying migraine risk loci, a recent analysis of three different imputation models showed a total of 62 putative novel migraine risk genes identifed at 32 independent genomic loci. 5 This evidence suggests the multifactorial nature of migraine.

There is no reason to expect a winner in the battle of genetics versus environment. To move further in the understanding of primary headaches, as an example of a complex, multifactorial disorder, we should identify subgroups of patients where a particular aspect is predominant, but keeping the idea where many factors contribute equally.
